# *RACGAP1* and *MKI67* are potential prognostic biomarker in hepatocellular carcinoma caused by HBV/HCV via lactylation

**DOI:** 10.3389/fonc.2025.1537084

**Published:** 2025-05-12

**Authors:** Muhammad Muddasar Saeed, Xinying Ma, Xinyu Fu, Ikram Ullah, Tanveer Ali, Changchuan Bai, Ying Liu, Chengyong Dong, Xiaonan Cui

**Affiliations:** ^1^ Department of Oncology, The First Affiliated Hospital of Dalian Medical University, Dalian, China; ^2^ Department of Biochemistry and Molecular Biology, College of Basic Medical Sciences, Dalian Medical University, Dalian, China; ^3^ Basics discipline of Integrated Traditional Chinese and Western Medicine, Dalian Medical University, Dalian, China; ^4^ Dalian Traditional Chinese Medicine Hospital, Dalian, China; ^5^ Department of Oncology, Affiliated Zhongshan Hospital of Dalian University, Dalian, China; ^6^ Division of Hepatobiliary and Pancreatic Surgery, Department of General Surgery, The Second Affiliated Hospital of Dalian Medical University, Dalian, China

**Keywords:** lactylation, immune infiltrations, bioinformatic analysis, HCV-HCC, MKI67, RACGAP1

## Abstract

**Introduction:**

Hepatocellular carcinoma (HCC) is recognized as the prime and lethal form of liver cancer caused by the hepatitis B virus (HBV) and hepatitis C virus (HCV) globally. Lactate is an end product of glycolysis that influences epigenetic expression through histone lactylation. While *MKI67* and RACGAP1 play crucial roles in HBV- and HCV-related HCC. However, the role of lactylation-related genes (LRGs) effects in this context remains unclear. This study innovatively explored the role of LRGs in HBV/HCV-associated HCC, identifying novel biomarkers for diagnosis and prognosis.

**Methods:**

The present study used various online databases for analysis, and the findings were validated via immunohistochemical (IHC) analysis of HCC patient samples (n=60).

**Results:**

We identified six signature LRGs (*ALB, G6PD, HMGA1, MKI67, RACGAP1*, and *RFC4*) possess prognostic potential, correlation with immune infiltration, and lactylation-related pathways, providing novel insights into tumor microenvironment (TME) of HCC. Moreover, *MKI67* and *RACGAP1* were significantly associated with HBV- and HCV-related HCC. IHC confirmed these findings, with high expression of *MKI67* and *RACGAP1* was significantly linked with HBV/HCV-associated HCC compared to non-viral HCC. The expression is also significantly associated with key clinical variables.

**Conclusion:**

Our results suggest that *MKI67* and *RACGAP1* could serve as promising biomarkers for detecting and predicting HCC caused by HBV/HCV via lactylation, opening a new direction for immune-targeted therapies.

## Introduction

Hepatocellular carcinoma (HCC) is the most common lethal form of liver cancer worldwide, accounting for 80% of liver cancers, and is the third leading cause of cancer-related deaths (1, 2). HCC typically affects individuals aged 30–50 years and is associated with various risk factors, such as hepatitis B or hepatitis C, alcohol abuse, smoking, obesity, and type 2 diabetes (1, 3). Notably, 80% of liver cancers are linked to viral infections (4). Despite multiple treatment options for HCC, such as surgery, transplantation, radiation, and chemotherapy, the 5-year survival rate is less than 20% (5). Therefore, it is crucial to find new biomarkers to support in diagnosis, improve survival, and monitor the reoccurrence of liver cancer. Investigating genes for HBV/HCV-related HCC via lactylation could enhance our understanding of the virus’s contribution to HCC development and potential therapeutic targets.

While the ability of HBV and HCV to affect liver cancer is well documented (6), the precise mechanisms by which these viruses contribute to HCC progression remain unclear. HBV infection leads to numerous health problems and fatalities associated with liver diseases such as HCC, cirrhosis, and liver decompensation (7). Recent estimates revealed that one in three liver cancer deaths are linked to HBV (8, 9). Chronic HBV infection is a prominent cause of death from liver cirrhosis and HCC, with approximately 0.82 million deaths annually (10). Despite widespread HBV vaccination, approximately 296 million cases were reported in 2019, and 1.5 million new infections are reported each year (11).

Numerous studies have shown that HCV infects approximately 17 million people annually, with approximately 71 million infections to date (12). If HCV is left untreated, it can lead to chronic viral infection, and approximately 20% of these patients develop liver cirrhosis. Once cirrhosis develops, 1–4% of patients progress to develop liver cancer each year (13). Additionally, 33% of people with HCV who do not have cirrhosis will also develop liver cancer within 30 years (14).

It is commonly believed that eradicating a viral infection can prevent the progression of virus-related cancer. Direct antiviral agents (DAAs) eliminate HCV, but liver cancer can still develop in individuals with advanced liver diseases. Recent research revealed an increase in liver cancer rates even after successful HCV eradication with DAAs (15). These findings reveal that removing the virus and infection treatment are insufficient to halt liver cancer development. To effectively understand and prevent cancer caused by viruses, it is crucial to study how viruses affect cellular processes, including cell growth, movement, and genetic changes (16). Identifying signature genes for HBV/HCV could provide a means for the early diagnosis and management of HCC.

Employing gene signatures at the mRNA level enhances the prognosis of individuals with HCC. Previous studies have demonstrated that gene signatures can predict liver cancer development (17, 18). Lactylation, a new approach to protein modification discovered in 2019, involves the addition of lactate molecules to lysine residues (19). The Warburg effect is characterized by increased anaerobic glycolysis and the production of lactic acid (20, 21). For example, researchers have reported that in liver cancer, the less efficient sugar-processing enzyme HK4 is replaced by the more efficient HK2, leading to increased glucose uptake by cancer cells (22). A 2023 study revealed that KIF2C (Kinesin Family Member 2C) is linked to *MKI67*, *RACGAP1*, *RFC4*, and STMN1 at the transcriptome level, suggesting that these genes might play a role in lactylation-related processes in HCC (23). This research also revealed that LRGs could be used to diagnose and treat HCC (23). Although researchers have identified the importance of specific genes in HCC (17, 24, 25), further studies are needed to explore the significance of LRGs, especially in HBV/HCV-induced HCC.

The impact of *MKI67* and *RACGAP1* on HCC linked to hepatitis B and C is a vital subject for research. *MKI67* is a recognized marker used to detect cellular proliferation (26, 27). It has been associated with genetic changes near the TTN and CCDC8 genes in HBV-related liver cancer (28). These changes could be useful for predicting patient outcomes. The overexpression of *MKI67* is linked to worse overall survival (OS) and increased reoccurrence rates in patients with HBV-related liver cancer (28). Recent studies have also revealed a connection between *MKI67* and HCV-related liver cancer. Certain microRNAs in naive T cells are connected to *MKI67* expression, suggesting that *MKI67* may play a part in liver cancer related to both HBV and HCV (29).

*RACGAP1* plays a pivotal role in cell division and cell cycle regulation. Recent studies have demonstrated that *RACGAP1* is a valuable marker for predicting outcomes and understanding the immune response in various cancers, including liver cancer (30). The overexpression of *RACGAP1* has been linked with poor prognosis and enhanced metastasis in multiple cancers, including HCC (31). For example, a 2015 study reported increasing the aggressiveness of tumors and facilitating lymph node metastasis in patients with colorectal cancer (32). Similarly, a study focusing on HCC revealed that *RACGAP1* interacts with HIF-1alpha, influencing hepatocarcinogenesis (33). These findings emphasize the multidirectional role of *RACGAP1* in cancer development and highlight its role as a therapeutic target.

Furthermore, recent studies have also underscored the role of lactylation, a novel epigenetic modification, in cancer progression. LRGs influence cancer biology, including immune infiltration and prognosis, in various cancer types (21, 34). For example, a multiomic study identified *RACGAP1* as one of the hub LRGs associated with poor prognosis and immune infiltration (21). Similarly, metabolic reprogramming is frequently observed involving alteration of fatty acid synthesis, glucose metabolism, and overall metabolic process in HCC (21). These studies suggest that lactylation could play a crucial role in modulating cancer progression.

This study identified a set of LRGs for HCC by using “The Cancer Genome Atlas Liver Hepatocellular Carcinoma (TCGA-LIHC)”, three “Gene Expression Omnibus (GEO)” datasets, and a previously published LRG list. We employed various bioinformatic tools to identify virus-related genes, which were validated via IHC analysis of human liver tissue samples. Furthermore, we investigated the relationships among the TME, immune infiltration, gene expression, and clinical variables. This study aimed to identify potential signature genes for HCC caused by HBV/HCV for screening and management. Our results indicate that *MKI67* and *RACGAP1* are significantly associated with immune infiltration and prognosis in HCC, suggesting their role as biomarkers for early diagnosis and targeted treatments.

## Materials and methods

### Data collection and identification of DEGs related to HCC

The mRNA transcriptome profiles and corresponding clinical information of 377 patients were downloaded from the TCGA database (https://protal.gdc.cancer.gov/) (34, 35). A scale method-based normalization approach was performed with the gene expression profiles via the R package “limma” (v3.60.4). This method was chosen because of its wide use in medical research, its robustness in handling RNA-seq data, and its ability to ensure that the normalized data are suitable for downstream analysis. The expression profiles of genes related to HCC were obtained from the GEO (36). Three GEO datasets, GSE14520 (37), GSE114564 (38), and GSE25097 (39) were selected to identify DEGs via GEO2R between HCC and nontumor samples (40). The cutoff for DEGs was set as |log2FC| > 1 and adjusted p-value < 0.05. These thresholds were chosen to ensure that the identified DEGs comprised a significant and biologically relevant change in expression levels.

To identify similar genes across all four datasets, the “VennDiagram” R package was used. This package was selected to facilitate the identification of genes that are common in different datasets (41).

### Expression, PPI, and functional enrichment analysis of DEGs

Volcano plots were created to visualize the relationships among the common DEGs via the “ggplo2” R package (42), with significance thresholds of p values <0.05 and |log2FC| >1. We constructed a protein-protein interaction (PPI) network and heatmap via the ‘igraph’ and ‘pheatmap’ R packages, respectively (43, 44). The PPI network was generated based on STRING-backed data, and a heatmap was designed by using expression data of DEGs across the datasets. For functional enrichment analysis, the “enrichplot” R package was employed for GO and KEGG pathway analysis. This analysis investigated the potential biological functions of the DEGs (45).

### Identification of lactylation-related DEGs via the LASSO model construction

Previously reported 330 LRGs were selected (23) to identify the LRGs from the common DEGs via the “VennDiagram” R package (41). This intersection helped us to identify genes common to both datasets. We employed the ‘ggplot2’ R package to visualize lactylation-related DEGs in volcano plots based on their expression (46). The PPI network was constructed via the ‘igraph’ package, with data retrieved from the STRING database (43). This network helped us visualize the linkages between proteins. Furthermore, the R package “enrichplot, clusterProfiler” was employed for Gene Ontology (GO) analysis to investigate the potential biological functions of lactylation-related DEGs (45). Finally, a correlation heatmap was created via the ‘pheatmap’ package between lactate accumulation genes and production-associated genes (LDHA, LDHB, EP300, and HIF1A) in the TME and LRGs (47). Moreover, to verify the prognostic significance of the LRGs in HCC, a predictive model was executed via least absolute shrinkage and selection operator (LASSO) regression via the “glment” R package. The risk score for each HCC patient was calculated (risk score = Σ (coefficient × gene expression)), where the coefficient and expression level (E) corresponded to each LRG. OS was compared between the high- and low-risk groups via Kaplan–Meier (KM) curves. Additionally, we conducted a correlation analysis between LRGs and widely recognized biomarkers (48).

### Gene expression profiling and survival analysis for LRGs

We validated the expression patterns of the identified lactylation-related DEGs via The University of ALabama at Birmingham CANcer data analysis Portal (UALCAN, http://ualcan.path.uab.edu/) (49) and the Gene Expression Profiling Interactive Analysis (GEPIA) web tool (http://gepia.cancer-pku.cn/ accessed on July 7, 2020) (50). These online tools provide comprehensive expression data across cancer types. Consequently, to assess overall survival (OS), disease-free survival (DFS), and progression-free survival (PFS), we utilized KM plotter (https://kmplot.com/ accessed in October 2023) (51, 52). This tool allows us to perform real-time survival analysis between high- and low-risk groups.

### Immune infiltration and pathway correlation analysis of the LRGs

Gene set cancer analysis (GSCA) was used to assess the correlation of methylation and pathways (inhibitors, activators) with the identified LRGs in liver cancer. This widely used platform has 7876 samples from 32 types of cancer, providing comprehensive cancer-related pathway analysis (53). Additionally, we investigated the correlation of LRGs with immune checkpoint inhibitors (ICIs). The R package “tidyverse” was used to prepare the data, and “ggplot2” was used to create a heatmap. Moreover, the TIMER tool was used to validate the relationships between the expression of six LRGs and immune cells across 40 cancer types from TCGA data (“https://cistrome.shinyapps.io/timer/) (51, 54) via the deconvolution method.

### Drug sensitivity to LRG mRNA expression

In addition to immune cell correlation, we conducted drug-gene interaction analysis for six LRGs via the Drug–Gene Interaction Database (DGIdb), which was accessed on Dec 21, 2023 (55). These data have been widely used in previous studies and consolidate data from various sources to demonstrate drug-gene interactions and gene categories. Furthermore, the Genomic of Drug Sensitivity in Cancer (GDSC) database was utilized to determine the correlation between the identified LRGs and drug sensitivity. This widely used platform has an IC50 of 265 molecules in 860 cell lines (56).

### Gene enrichment and virus interactions analysis

This study investigated six LRGs and oncoviruses associated with liver cancer via the OncoDB database (https://oncodb.org/, Accessed on Feb 12, 2024). A p-value>0.05 was considered to indicate statistical significance. Furthermore, gene set enrichment analysis (GSEA) was performed via the CAMOIP (www.camoip.net) web tool. This web tool facilitated the elucidation of the biological pathways and mechanisms associated with these genes.

### Immunohistochemical staining

Our comprehensive bioinformatic analysis of the six LRGs revealed that *MKI67* and *RACGAP1* were significantly associated with HBV and HCV in HCC. We conducted an immunohistochemical study of the patient’s liver tissue to validate these findings. This study was conducted after approval from the Institutional Review Board of the First Affiliated Hospital of Dalian Medical University (Approval number: “PJ-KS-KY-2018-07 (X)”). Written informed consent was obtained from all participants. All methods were conducted in accordance with the Declaration of Helsinki. Liver tissue samples were collected from 60 individuals with HCC, including 20 individuals diagnosed with HCC without infection, 20 with HBV, and 20 with HCV infection. The etiological information for selected HCC samples without infection included 17 males and 03 females (n=20), while for HCC samples with HBV, 17 males and 03 females (n=20) were included, and for HCC samples with HCV, 16 males and 4 females (n=20) were included. Detailed clinical information on these patients is given in **Table 1**.

**Table 1:** Etiological and demographic information of the HCC samples.

*HCC without viruses*				HCC with HBV				HCC with HCV			
*Gender*	Age	Clinical Stage (CNLC)	Pathological Grade	Gender	Age	Clinical Stage (CNLC)	Pathological Grade	Gender	Age	Clinical Stage (CNLC)	Pathological Grade
*Female*	58	Stage Ia	Grade III	Male	49	Stage Ia	Grade IV	Male	63	Stage Ia	Grade II
*Male*	68	Stage Ia	Grade II	Male	64	Stage Ia	Grade III	Male	78	Stage Ia	Grade IV
*Male*	52	Stage IIIa	Grade IV	Male	57	Stage Ia	Grade II	Female	75	Stage Ia	Grade II
*Male*	69	Stage Ia	Grade II	Female	51	Stage Ia	Grade II	Male	53	Stage Ia	Grade II
*Male*	55	Stage Ia	Grade IV	Male	41	Stage Ia	Grade II	Male	50	Stage Ia	Grade II
*Male*	68	Stage Ia	Grade I	Male	52	Stage Ib	Grade IV	Male	67	Stage Ib	Grade II
*Male*	46	Stage Ib	Grade III	Female	63	Stage IIIa	Grade III	Female	63	Stage Ib	Grade II
*Male*	63	Stage Ia	Grade II	Male	58	Stage IIa	Grade II	Male	61	Stage Ib	Grade II
*Male*	69	Stage Ia	Grade I	Male	48	Stage IIIb	Grade IV	Male	65	Stage Ia	Grade II
*Male*	70	Stage IIIa	Grade II	Male	64	Stage Ia	Grade II	Male	73	Stage Ib	Grade II
*Male*	37	Stage IIIa	Grade IV	Male	59	Stage Ia	Grade II	Male	57	Stage Ia	Grade II
*Female*	61	Stage Ib	Grade III	Male	65	Stage Ia	Grade III	Male	59	Stage Ib	Grade II
*Male*	55	Stage Ib	Grade II	Male	66	Stage Ib	Grade I	Male	60	Stage Ia	Grade II
*Male*	56	Stage Ia	Grade II	Male	56	Stage Ia	Grade II	Male	74	Stage II	Grade II
*Male*	56	Stage IIIa	Grade II	Female	62	Stage IIa	Grade II	Female	69	Stage Ia	Grade II
*Male*	67	Stage Ia	Grade I	Male	46	Stage Ib	Grade III	Male	76	Stage Ia	Grade II
*Male*	63	Stage Ib	Grade II	Male	52	Stage IIa	Grade II	Male	66	Stage IVb	Grade II
*Female*	45	Stage IIIa	Grade II	Male	61	Stage Ia	Grade I	Male	70	Stage IIIa	Grade II
*Male*	67	Stage Ib	Grade I	Male	50	Stage Ia	Grade III	Male	68	Stage II	Grade II
*Male*	54	Stage Ia	Grade II	Male	69	Stage Ia	Grade I	Female	74	Stage IVb	Grade II

Immunohistochemical staining was conducted as described in previous studies (57, 58). Briefly, formalin-fixed, paraffin-embedded tissues were removed and mounted on glass slides. The sections were deparaffinized with xylene and rehydrated through a series of ethanol dilutions (100% to 70%). Antigen retrieval was performed via citrate buffer (pH 6.0) in a pressure cooker for 10 minutes with proper heating (90–95°C). Endogenous peroxidase activity was quenched with 3% hydrogen peroxide in methanol for 30 minutes at room temperature.

The sections were then blocked with 3% BSA (Sigma–Aldrich, St. Louis, MO, USA) at room temperature for 30 minutes to prevent nonspecific binding. The sections were incubated with primary antibodies against *MKI67* (1:100 dilution, Ki67 rabbit mAb (A20018) and *RACGAP1* (1:100 dilution, *RACGAP1* rabbit mAb (A24948), ABclonal) overnight at 4°C. After being washed with PBS, the sections were incubated with an HRP-labeled goat anti-rabbit IgG (1:200; GB23303; Servicebio) secondary antibody for 30 minutes at room temperature. The immunoreaction was visualized via 3,3’-diaminobenzidine (DAB) counterstaining with hematoxylin. The sections were washed, and the slices were dehydrated with alcohol and cleared in xylene. Images were captured via a light microscope (LEICA DM 2500) at 40x magnification. The expression levels of *MKI67* and *RACGAP1* were quantified via the optical density (OD) method by two independent scholars. Five random high-power fields (40×) were selected for each section, and the OD values were measured using ImageJ software (NIH, USA).

### Statistical analysis

Statistical analyses were performed using R software (version 4.4.1). The normality of data distribution was assessed using the Shapiro–Wilk test and one-way Analysis of Variance (ANOVA) to compare OD values across different conditions for each gene. A p-value < 0.05 was considered statistically significant.

## Results

### Comprehensive analysis to identify differentially expressed genes associated with HCC

**Figure 1:**
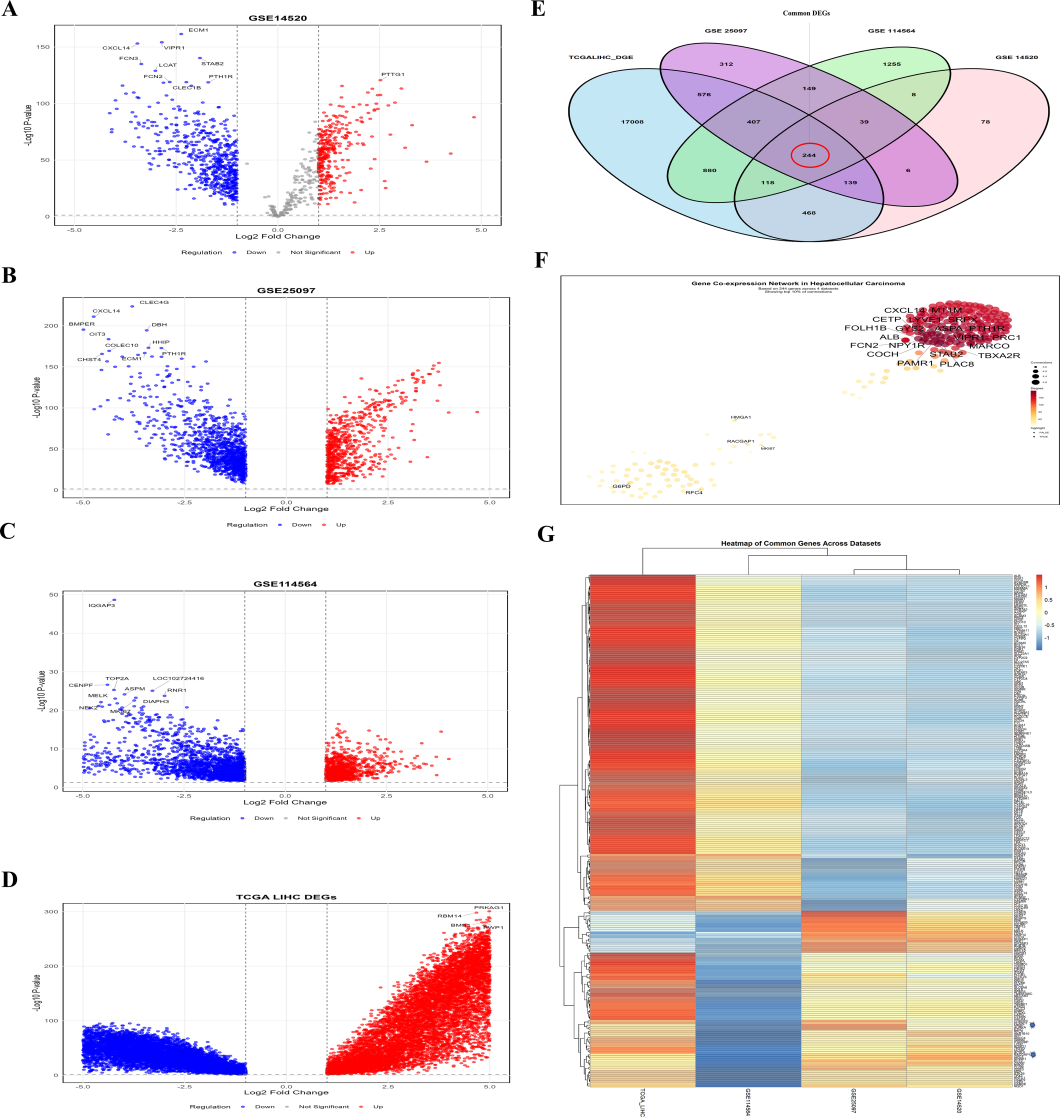
Comprehensive analysis of DEGs across the selected datasets **(A-D)** Volcano plots indicate the DEGs in HCC compared to normal liver tissue in the GSE14520, GSE25097, GSE114564, and TCGA-LIHC, respectively **(E)** Venn diagram illustrating the overlap of DEGs across the selected datasets **(F)** PPI network of the common DEGs **(G)** Heatmap showing the expression patterns of all 244 common DEGs across the four datasets. This analysis revealed that the common DEGs are interlinked and have different expressions across the dataset.

We identified DEGs from TCGA-LIHC and GEO datasets via the “limma” R package and the GEO2R analysis tool. The limit for DEGs was set at a log2-fold change (log2FC > 1) and a p-value < 0. 05). The TCGA-LIHC dataset identified 19840 DEGs; subsequently, the GEO datasets (GSE14520, GSE114564, and GSE25097) identified 1100, 3100, and 1872 DEGs, respectively (**Supplementary File-S1**). The volcano plots (**Figures 1A-D**) represent the gene expression variations in liver cancer, with red dots indicating upregulated genes, blue dots representing downregulated genes, and gray dots representing genes whose expression was not significantly altered. The x-axis shows the log2-fold change, and the y-axis shows the -log10(adjusted p-value). These visualization provide a summary of significant expression changes in dataset of HCC. Moreover, the “VennDiagram” R package was used to identify common genes, and we identified 244 common DEGs across all selected datasets (**Figure 1E**; **Supplementary File-S2**). **Figure 1F** shows a plot illustrating the interactions of the DEGs, which indicate the connection of LRGs. This PPI interaction indicate the functional relationships between DEGs in HCC. Finally, the heatmap (**Figure 1G**) visually compares gene expression across the datasets, with red indicating upregulated genes, blue indicating downregulated genes, and white indicating genes with minimal expression. This heatmap helps us compare how genes are expressed in different studies in HCC. These findings indicate that the identified DEGs might be crucial for identifying potential biomarkers of lactylation and targets for the treatment of HCC.

### Functional enrichment and pathway analysis of DEGs associated with HCC

**Figure 2:**
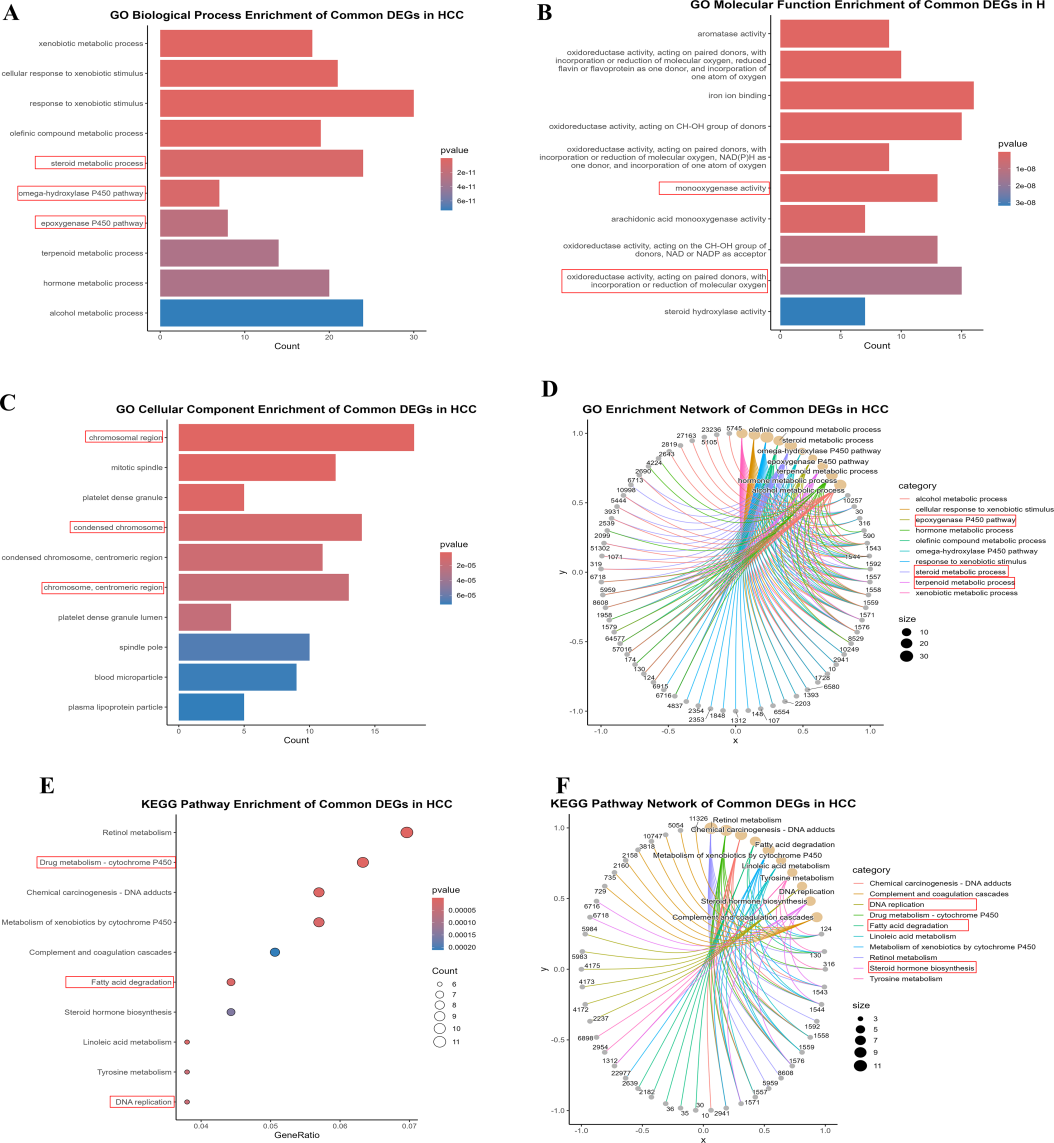
The enrichment analysis of 244 common DEGs **(A-C)** Bar plots illustrating the Gene Ontology, BP, MF, and CC, respectively **(E)** Dot plot indicating the KEGG pathways enrichment of DEGs **(D, F)** Circular network plots illustrate the GO and KEGG enrichments of DEGs. These visualizations underscore the relationship of DEGs with metabolic pathways.

Our previous findings of **Figure 1** explicated the relationships between common DEGs via PPI and differential expression. To further explore the significance of DEGs, GO and KEGG analyses were used to elucidate the role of DEGs in HCC progression and development. We selected the top ten significant GO terms and KEGG pathways (p values < 0.05). The DEGs were involved mainly in metabolic processes, including the xenobiotic metabolic process, the cellular response to xenobiotic stimulus, the olefinic compound metabolic process, and the steroid metabolic process (**Figures 2A-C**). These findings revealed the involvement of metabolic pathways in HCC development, which is relevant to lactylation process. Furthermore the KEGG analysis revealed that the DEGs are significantly involved in various metabolism-related pathways (**Figure 2E**), such as fatty acid degradation, drug metabolism, etc. In the bar and bubble plots, the x-axis shows the gene ratio, and the color indicates the adjusted p-value, whereas the y-axis is labeled with enrichment terms and KEGG pathways. The network plots (**Figure 2D, F**) depict node size as the gene count, edge thickness as the term overlap, and node color as the significance (darker = lower p-value). These analyses revealed key biological processes, cellular components, molecular functions, and pathways potentially involved in HCC development and progression, which is relevant to our study. **Table 2** lists the top five GO terms, which include B.P., CC, and M.F. (**Supplementary-S3**). Collectively, these findings highlight the involvement of DEGs in metabolic processes, suggesting their importance for lactylation in HCC.

**Table 2:** The top five GO phrases, which include Biological processes, Cellular components, and Molecular functions.

Ontology	ID	Description	p-value	Count
**BP**	GO:0006805	Xenobiotic metabolic process	3.02797E-14	18
	GO:0071466	Cellular response to xenobiotic stimulus	5.90207E-14	21
	GO:0009410	Response to xenobiotic stimulus	7.23169E-14	30
	GO:0120254	Olefinic compound metabolic process	2.25362E-13	19
	GO:0008202	Steroid metabolic process	5.28374E-12	24
**CC**	GO:0098687	Chromosomal Region	2.43467E-06	18
	GO:0072686	Mitotic Spindle	2.87564E-06	12
	GO:0042827	Platelet Dense Granule	4.52573E-06	5
	GO:0000793	Condensed Chromosome	9.97887E-06	14
	GO:0000779	Condensed Chromosome, Centromeric Region	1.46027E-05	11
**MF**	GO:0070330	aromatase activity	2.4623E-11	9
	GO:0016712	oxidoreductase activity, acting on paired donors, with incorporation or reduction of molecular oxygen, reduced flavin or flavoprotein as one donor, and incorporation of one atom of oxygen	1.5816E-10	10
	GO:0005506	iron ion binding	1.7149E-10	16
	GO:0016614	oxidoreductase activity, acting on the CH-OH group of donors	2.7362E-10	15
	GO:0016709	oxidoreductase activity, acting on paired donors, with incorporation or reduction of molecular oxygen, NAD(P) Has one donor, and incorporation of one atom of oxygen	1.1207E-09	9

### Identification and analysis of lactylation-related genes and the protein-protein interaction network associated with HCC

**Figure 3:**
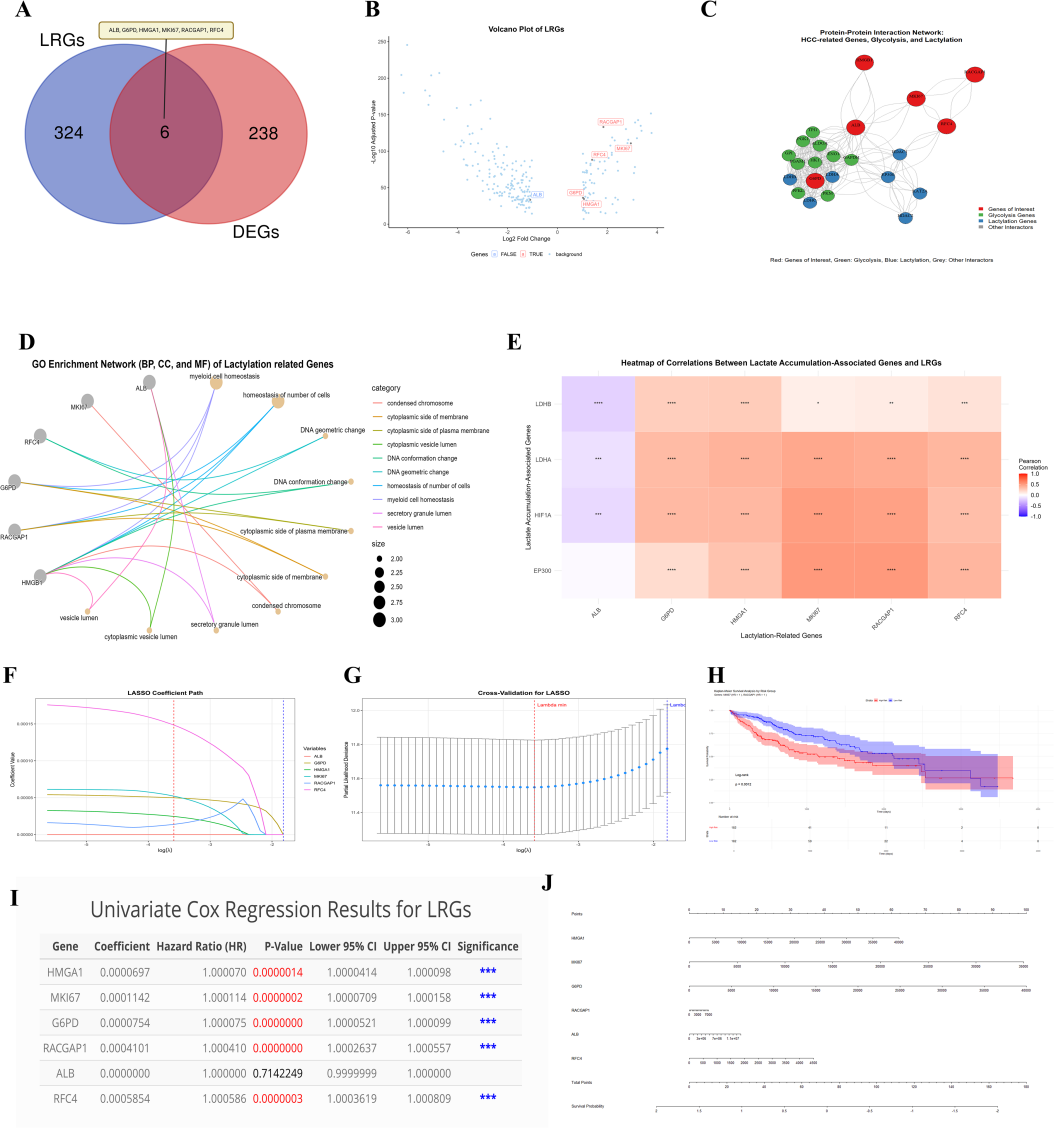
Comprehensive analysis of Lactylation-related genes. This figure shows a detailed analysis of LRGs in HCC, their interactions, expression profile, and roles in HCC progression **(A)** Venn diagram shows the overlap between 244 DEGs and 330 LRGs, overlapping identifies 06 common genes **(B)** Volcano plot displays the expression level of six common lactylation-related DEGs **(C)** The network plot shows interactions between six LRGs with glycolysis proteins. Red nodes: genes of interest; Green nodes: glycolysis-related genes; Blue nodes: lactylation-related genes **(D)** GO enrichment network for the six LRGs **(E)** Correlation heatmap of LRGs and lactylation accumulations associated genes in HCC **(F-J)** Plots showing the LASSO, Prognostic and risk score model of six LRGs. These analyses endorse that these LRGs could be potential prognostic markers in HCC except *ALB*. Statistical significance is indicated as follows: *p < 0.05; **p < 0.01; ***p < 0.001; ****p < 0.0001.

A comprehensive analysis was conducted to identify LRGs in HCC, integrating multiple analytical approaches. The “VennDiagram” R package was used to identify the genes common to the DEGs (244) and LRGs (330): *ALB*, *G6PD*, *HMGA1*, *MKI67*, *RACGAP1*, and *RFC4* (**Figure 3A**). We visualized the expression of six LRGs through a volcano plot by using ‘ggplot2’ (**Figure 3B**), where red highlights indicate upregulated genes and blue highlights indicate downregulated genes. The results revealed that five genes were upregulated, whereas *ALB* was downregulated. Taken together, the PPI networks (**Figure 3C**) revealed intricate connections between lactylation genes and other proteins, particularly those involved in glycolysis, suggesting a potential link to altered metabolism in HCC. The analysis revealed that these six LRGs are closely related to several glycolysis-related proteins (e.g., *PFKL, PKM, PGAM1, ALDOA, and GAPDH)* and other LRGs (e.g., *LADHA, LADHB, and LADHC)*. Literature analysis plots (**Supplementary-S7-I**) revealed that *MKI67, RACGAP1*, and *RFC4* have limited publications, suggesting their potential innovation and distinctiveness in HCC therapy.

### Functional enrichment and correlation analysis of the genes related to lactylation in HCC

We performed enrichment analysis on the six lactylation-related DEGs to explore their potential in the progression of HCC via R software. The results revealed that these genes were enriched mainly in cell cycle-related terms, including condensed chromosomes, the cytoplasmic side of the membrane, DNA conformation changes, and myeloid cell homeostasis (**Figure 3D**). These enrichments are known to be involved in the regulation of energy metabolism and have been implicated in cancer development. The correlation heatmap (**Figure 3E**) further illustrates the interrelationships between the identified lactylation-associated genes and genes associated with lactylation accumulation in HCC. Overall, this multifaceted analysis underscores the potential significance of lactylation-associated genes in HCC pathogenesis, particularly concerning the regulation of energy processes, and provides a foundation for future investigations into their roles in cancer development and potential therapeutic targeting.

### Construction and validation of the prognostic model for HCC using TCGA-LIHC data

Furthermore, the LASSO model was employed to identify prognostic markers among the LRGs (**Figure 3F**). The results revealed that *ALB* had the lowest coefficient value, whereas *RFC4* had the highest coefficient value, with the others also showing significant values. These findings suggest that these genes could potentially play a role in predicting patient prognosis. Subsequently, cross-validation for LASSO was represented by a lambda plot (**Figure 3G**). The KM plot revealed significant differences in OS between the high- and low-risk groups for *MKI67* and *RACGAP1*, with a p-value <0.05 (**Figure 3H**). Additionally, a univariate Cox regression analysis (**Figure 3I**) confirmed the significance of all the LRGs except for *ALB*. Finally, the nomogram model results illustrated (**Figure 3J**) the predictive power of the identified LRGs. To further validate these findings, we compared our LRGs with five widely used biomarkers (GPC3, HSP70, GP73, OPN, and AFP) reported in recent studies (48). Our results revealed that *MKI67* was significantly positively correlated with GPC3 and AFP, whereas *RACGAP1* was significantly positively associated with OPN and GPC3. Both *MKI67* and *RACGAP1* were significantly negatively correlated with GP73 (**Supplementary S6**). Collectively, these results suggest that LRGs may serve as robust prognostic markers in HCC.

### Validation of expression, stage-specific expression and survival analysis of LRGs in HCC

**Figure 4:**
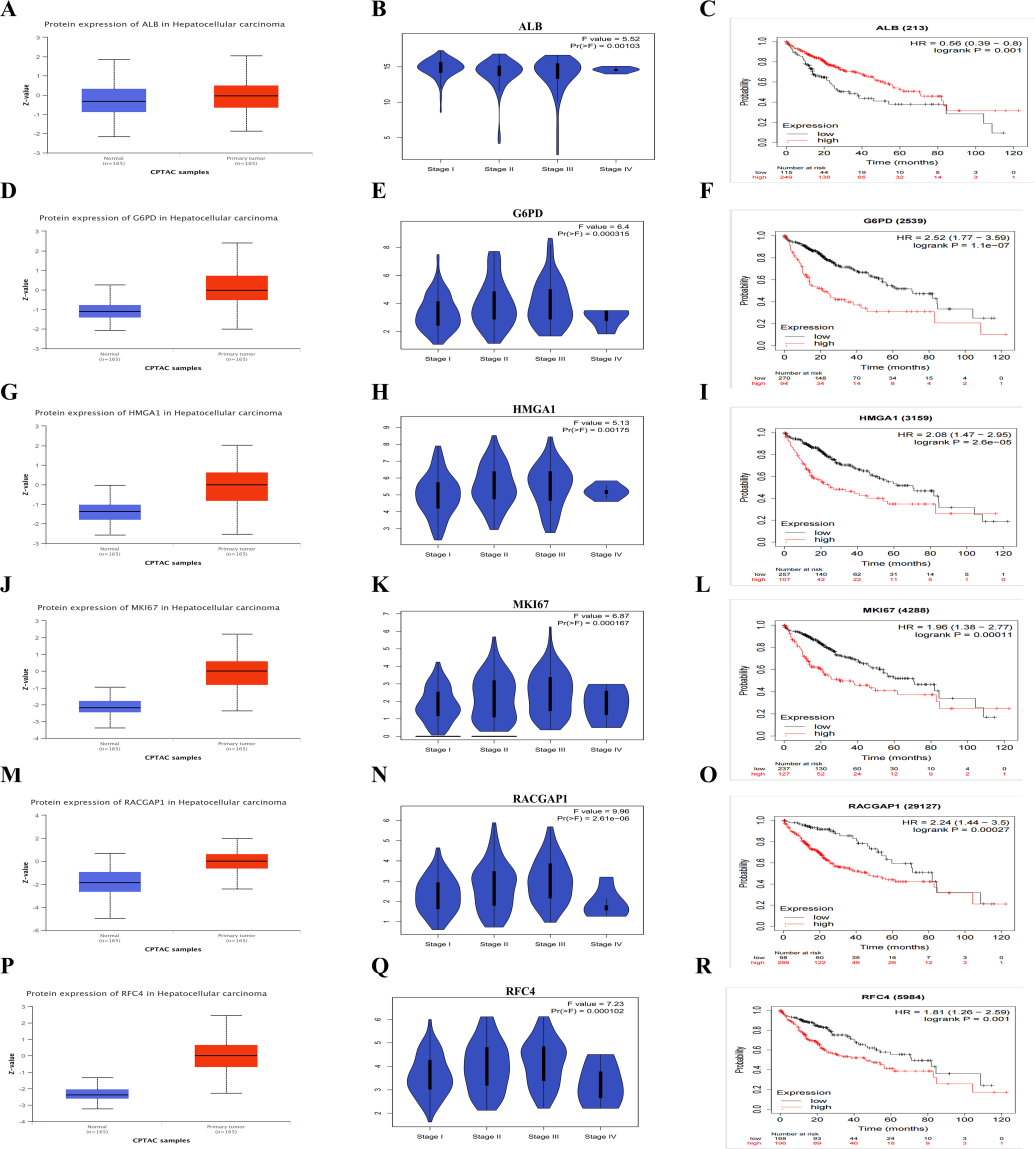
The Expression and Survival Analysis of Six LRGs **(A, D, G, J, M, P)** The box plots indicate the expression levels of six LRGs between HCC tumor vs normal liver tissue **(B, E, H, K, N, Q)** The violon plots illustrate the expression levels between stages **(C, F, I, L, O, R)** The Kaplan-Meier plots showing the overall survival plot between low and high-risk groups.

We conducted the expression analysis of six LRGs in both normal and primary tumor samples of HCC. *ALB* was significantly lower in primary tumors compared to normal samples (p <0.05). The expression levels of *G6PD*, *HMGA1*, *MKI67*, *RACGAP1*, and *RFC4*, were significantly higher in tumor samples with (p < 0.05) (**Figures 4A, D, G, J, M, P**). The blue violin plots represent the distribution the frequency of expression levels, across different HCC stages (**Figures 4B, E, H, K, N, Q**). F values and corresponding p values from ANOVA are provided in the plots. Moreover, we investigated the OS of LRGs with both low and high expression levels (**Figures 4C, F, I, L, O, R**). The log-rank p values were < 0.05, with hazard ratio (HR) values of 2.52 (1.77–3.59), 2.08 (1.47–2.95), 1.96 (1.38–2.77), 1.96 (1.38–2.77), and 1.81 (1.26–2.59), respectively. Thess findings revealed the differential expression of LRGs between normal and tumor tissues, various stages and their association with overall survival. These results underscoring the potential of LRGs as prognostic biomarker in HCC.

Similarly, we also investigated the PFS and recurrence-free survival (RFS) suggesting their involvement in HCC progression and development. High expression of *G6PD*, *HMGA1*, *MKI67*, *RACGAP1* associated with these LRGs at both low and high expression levels (**Supplementary-S4-A, B**). These findings highlight their specific potential as markers for HCC diagnosis and prognosis.

### Analysis of immune infiltration and pathways associated with LRGs in HCC

**Figure 5:**
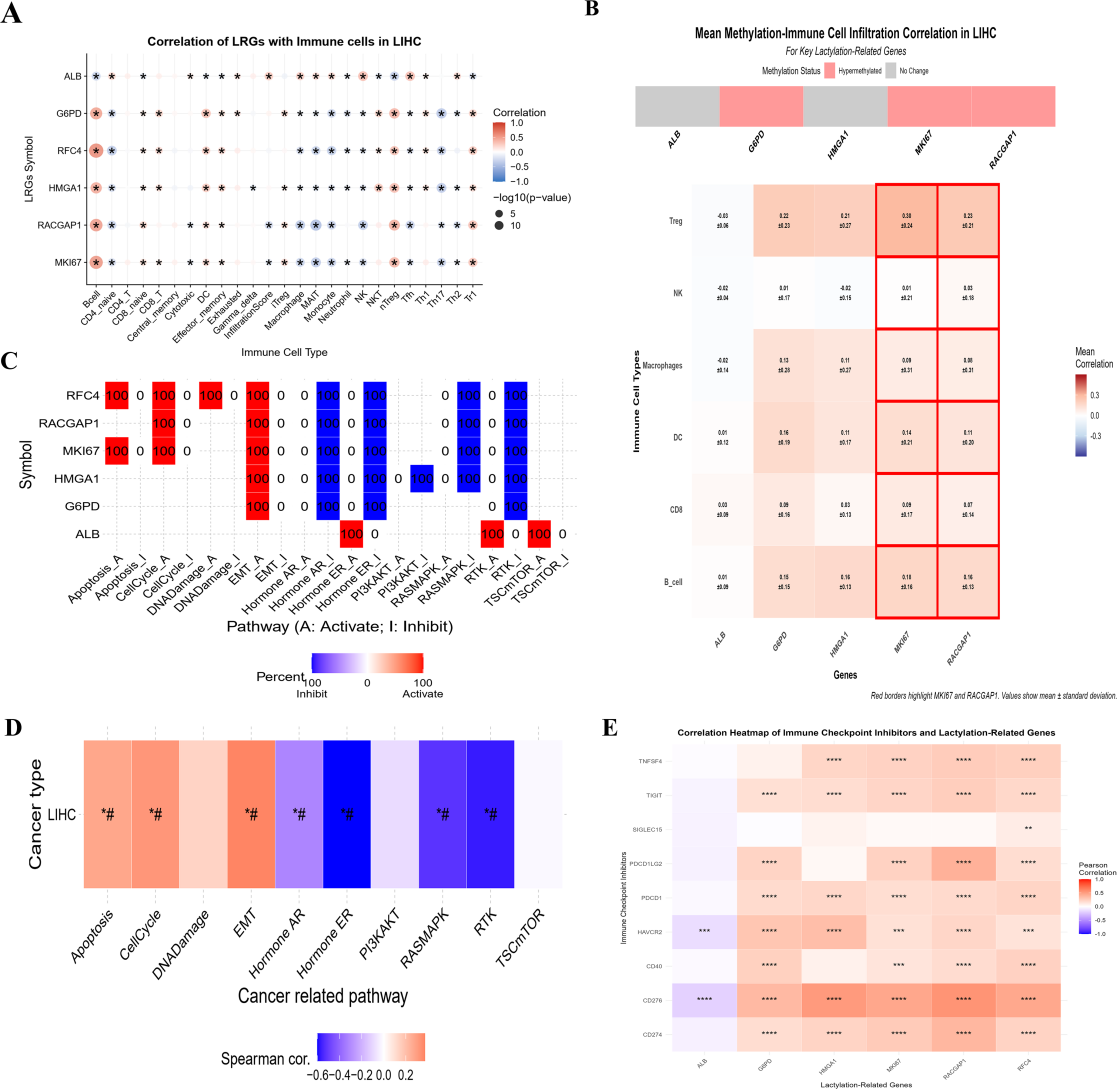
The correlation of LRGs and immune infiltration **(A)** The individual heatmaps indicating the correlation between LRGs and immune cell infiltration across the 40 types of cancers **(B)** The bar plot showing the correlation between LRGs and immune cells in liver cancer. **(C, D)** The bar plots showing the correlation between LRGs and pathways involved in liver cancer **(E)** The Pearson correlation representing by heatmap between six LRGs with ICIs. These analyses indicate the relationship of LRGs in HCC progression. Statistical significance is indicated as follows: *p < 0.05; **p < 0.01; ***p < 0.001; ****p < 0.0001.

By using the web tool “GSCA”, we investigated immune infiltration and methylation with the expression of six LRGs in liver cancer. The results revealed a significant correlation between these LRGs and various immune cells, such as B cells, Tregs, DCs, macrophages, and myeloid dendritic cells, in liver cancer (**Figure 5A**). The dot plot shows a remarkable positive correlation with Tregs and B cells and a negative correlation with macrophages, monocytes, NK cells, etc. Red indicates a positive correlation with immune cells, whereas blue indicates a negative correlation, with significant stars. The heatmap depicted the mean correlation between methylation and immune infiltration of the six LRGs in liver cancer (**Figure 5B**), including a significant association. These results indicate that significant positive and negative correlations between these genes may indicate aberrant histone methylation activity, which could be crucial in cancer development.

To further elucidate the pathways involved, the findings highlighted that these LRGs are significantly correlated with cancer-related pathways, such as apoptosis, the cell cycle, and DNA damage (**Figures 5C, D**). Additionally, correlation analysis between the LRGs and ICI using TCGA-LIHC data (**Figure 5E**) indicated that the five genes were significantly positively correlated with ICI, whereas *ALB* was negatively correlated. Collectively, these findings suggest that the six LRGs could be potential biomarkers, highlighting their role in the TME, which could be important for developing targeted immunotherapy in HCC. Additional informative analysis of immune cells with LRGs via TIMER data is shown in **Supplementary Figure S5**.

### Exploring drug-gene interactions of lactylation-related genes in HCC

Our results from gene-drug interactions revealed 163 drug interactions, with 81 drugs approved by the Food and Drug Administration (FDA) with three genes (*RACGAP1*, *G6PD*, and *ALB*) shown in **Supplementary S6A**. Further analysis revealed that 13 of these approved drugs are also related to immunotherapy. Moreover, the GDSC platform was used to determine the correlation between mRNA expression and drug sensitivity in cancer (**Supplementary Figure S6B, C**). The results indicated that most of the identified LRGs are negatively correlated with drug sensitivity, except *G6PD*. These findings emphasize the potential and clinical importance of the identified LRGs in HCC therapies.

### Integrated expression profile with OncoDB and GSEA of lactylation-related genes in HCC

**Figure 6:**
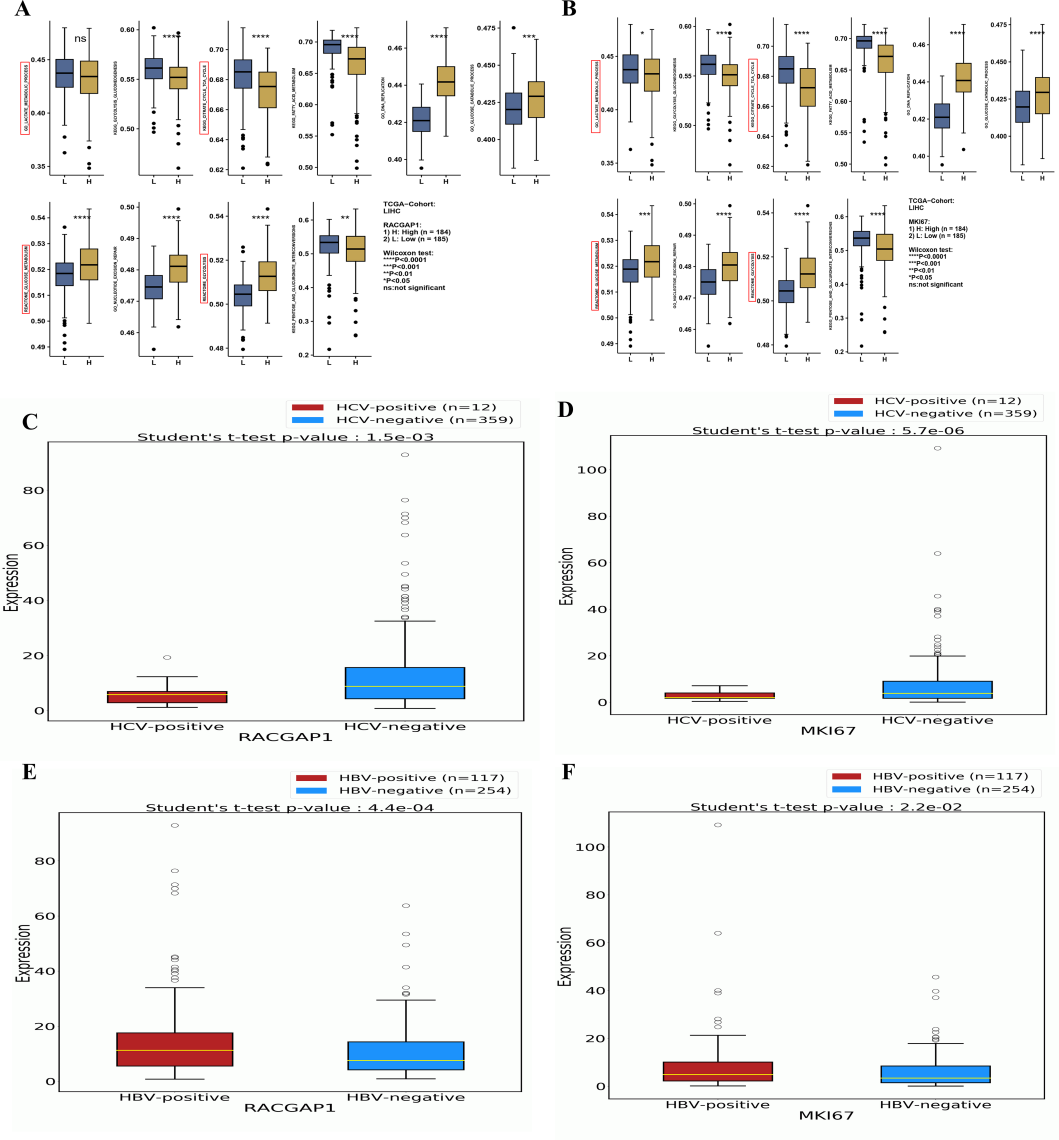
A comprehensive Correlation Analysis Between LRG Expressions Enrichments and Viruses **(A, B)** Box plots demonstrating the most relevant enrichments of *MKI67* and *RACGAP1* between high vs low expression in liver cancer **(C, D)** The box plot showing the significant correlation of these genes with HCV-associated HCC **(E, F)** The box plot showing the significant correlation of these genes with HBV associated HCC. Statistical significance is indicated as follows: ns (Non-significant) p > 0.05; *p < 0.05; **p < 0.01; ***p < 0.001; ****p < 0.0001.

Furthermore, GSEA was conducted for *MKI67* and *RACGAP1* by dividing the gene profile into high-expression groups and low-expression groups. Box plots represent the 10 enriched processes, which are related mostly to the regulation of energy in cancer, such as the lactate metabolic process, KEGG glycolysis, and the cell cycle (TCA) (**Figures 6A, B**). The expression profile of six LRGs were analyzed via “OncoDB”. The results revealed significant correlations between *MKI67* and *RACGAP1* with both HCV-HCC (p < 0.05) and HBV-HCC (p > 0.05) (**Figures 6C-F**). Aditionally, *RFC4* also demonstrated a robust correlation with HBV-HCC (p < 0.05).

Althogh the *MKI67* and *RACGAP1* were significantly differentially expressed between positive and negative HCV, but we observed the higher median of expression in HCV-negative compared to the HCV-positive patients. This discrepancy maybe due to the attributed disparity in sample sizes beween the groups. To validate these findings we conducted external validataion. The two HCV-related datasets (GSE140845, and GSE154211) were selected frome GEO database. The analysis employed between HCV-positive v.s HCV-negative HCC samples (**Supplementary-S7**, **S8**). The results confirmed that all the HCV-related HCC has high expression values of *MKI67* and *RACGAP1* as compared to non-viral samples.

These findings suggest that these genes may play critical roles in the progression of HBV/HCV-induced HCC. The other genes (*G6PD*, *HMGA1*, and *ALB*) presented weaker associations with HBV/HCV-induced HCC, indicating that their roles might be more general in HCC pathogenesis than specific to viral etiology (**Supplementary-S8**). These results collectively highlight the potential of LRGs as biomarkers and therapeutic targets in HCC, particularly in viral hepatitis-associated HCC. The identified drug interactions and differential expression patterns provide a foundation for further investigations into novel treatment strategies for HCC.

### Immunohistochemical validation of *MKI67* and *RACGAP1*

**Figure 7:**
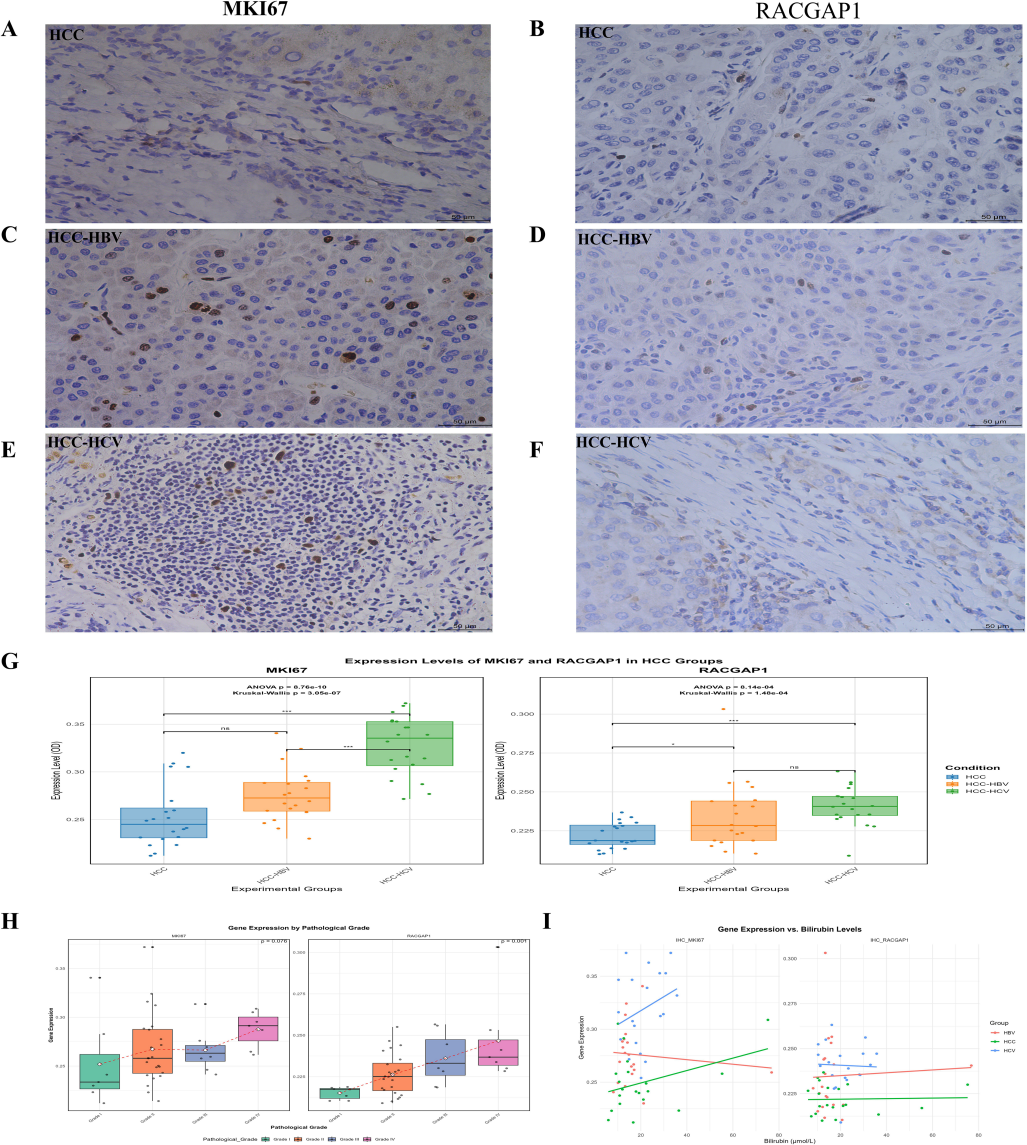
Validation of *MKI67* and *RACGAP1* expression levels in HCC groups by IHC **(A, C, E)** The images representing the expression level of *MKI67* between HCC, HCC-HBV and HCC-HCV, similarly **(B, D, F)** images showing the *RACGAP1* expression between HCC, HCC-HBV and HCC-HCV **(G)** The box plots indicating the OD values of these genes across the HCC groups **(H, I)** The correlation of *MKI67*, *RACGAP1* expression with pathological grade and bilirubin levels indicating by box plot and scatter plot, respectively. Statistical significance is indicated as follows: ns (non-significant) p > 0.05; *p < 0.05; **p < 0.01; ***p < 0.001.

We collected HCC patient samples (n=60), including 20 from patients with HCC without the virus, 20 from patients with HBV-HCC, and 20 from patients with HCV-HCC. The results of the “ANOVA” revealed a significant effect of condition on the OD values of both genes. *MKI67* p < 0.05, and *RACGAP1*, p < 0.05. Pairwise t-tests with Bonferroni correction for *MKI67* indicated significant differences between HCC, HBV-HCC, and HCV-HCC patients (p < 0.05) (**Figures 7A, C, E**). For *RACGAP1*, significant differences were observed between HCC, HBV, and HCV (p < 0.05) (**Figures 7B, D, F**).

The Kruskal–Walli’s test confirmed the significant effects of condition on the OD values for both *MKI67* (χ² (2) = 30.004, p < 0.05) and *RACGAP1* (χ² (2) = 17.630, p < 0.05). Dunn’s test for *MKI67* also revealed significant differences between HCC, HBV, and HCV (p = 0.05). For *RACGAP1*, a significant difference was found between HCC, HBV, and HCV (p < 0.05), whereas differences between HBV and HCV (p = 0.79) were not significant boxplots (**Figure 7G**). Our results revealed that patients diagnosed with HCV had much higher expression levels of *MKI67* and *RACGAP1* than did patients diagnosed with HBV or HCC. *MKI67* levels varied across all groups, whereas significant differences in *RACGAP1* were observed only between the HCV-HCC and HBV-HCV groups. There was a notable association between pathological grade and *RACGAP1* (p=0.001), but for *MKI67*, it was not significant (p=0. 07). These findings highlight the differential expression of *MKI67* and *RACGAP1* in HCC, HBV, and HCV (**Figure 7H**) and provide valuable insights into the molecular mechanisms underlying these diseases. A marked difference in bilirubin levels was observed between the groups (p=0.0424) for both genes (**Figure 7I**).

## Discussion

Recent studies have highlighted the potential of lactate and its lactylation modification in cancer progression (59). Lactylation is a post-translational modification of proteins, plays a pivotal role in cellular metabolism, and is increasingly recognized for its involvement in the development of tumors (19, 60). Previous research indicated that the accumulation of lactate in tumors allows the cancer cells to evade the immune system, facilitating their growth and proliferation (61, 62). To elucidate the function of lactylation modification in HCC, we identified common genes between lactylation-related gene set and DEGs as LRGs. These six LRGs (*ALB*, *G6PD*, *HMGA1*, *MKI67*, *RACGAP1*, and *RFC4*) could serve as important markers for HCC. Our bioinformatic investigation indicated a significant association between *MKI67* and the *RACGAP1* gene in HCC linked to HBV and HCV. Further validation through IHC analysis of patient samples confirmed that these two genes are more strongly correlated with HCC caused by HBV/HCV compared to HCC without viruses. These findings underscore the significance of further investigating the potential of *MKI67* and *RACGAP1* in predicting outcomes and developing targeted therapies for HCC induced by viruses.

Chronic HBV/HCV infections are prevalent worldwide, with approximately 80% of liver cancer cases (63, 64). HCV is an oncogenic virus that promotes carcinogenesis through cycles of damage and regeneration, driven by molecular mechanisms, including inflammation, proliferation, apoptosis, and genomic alterations (14, 65). Our findings suggest that HCC associated with HCV may exhibit distinct characteristics, particularly in genes linked to lactylation. This emphasizes the importance of identifying specific for predicting disease progression and potential treatment targets, such as viruses-induced HCC.

Recent studies have shown, *RACGAP1* expression is correlated with tumor size, clinical grade, histological type, and prognosis in various tumor types. For example, it promotes cell motility and invasion in uterine carcinosarcoma, with its positive expression associated with poorer prognoses (66). Colorectal cancer patients have poor prognoses linked to *RACGAP1* expression (67). Another study reported that the overexpression of *RACGAP1* also predicts survival rates for squamous cell carcinoma (68). Consistent with these studies, this study also revealed that *RACGAP1* is a significant prognostic marker in HCC induced by HBV/HCV via lactylation. These collective findings underscore the importance of *RACGAP1* in HCC progression and prognosis.

Previous studies have shown that *MKI67* affects immune infiltration and T-cell fatigue and serves as a prognostic biomarker in cancers, especially HCC. Measuring *MKI67* levels improves the effectiveness of anti-LIHC immunotherapy by assisting in prognosis prediction (69). Our study also identified the links between clinical indicators, such as increased levels of bilirubin linked to liver dysfunction, and gene activity in patients with HCV (70). Additional comprehensive studies are required to confirm these outcomes and evaluate their clinical applications. This might assist in establishing potentially improving diagnostic and prediction of liver diseases.

The TME plays is pivotal in cancer progression, with lactylation playing a significant role within it (47). In this research, we identified LRGs to predict prognosis in HCC. The results indicated a significant correlation between LRGs and immune cells, demonstrating a positive correlation with, B-cells, Tregs, and Tr1 in LIHC while showing a negative correlation with, macrophages, th17 NK cells, etc. The pathways analysis further supported these findings and underscored the role of immune infiltration in HCC development. These results align with a broader understanding of TME’s role in cancer development and underscore the crucial role of LRGs as biomarkers for immunotherapy strategies in HCC.

Additionally, the correlation with selected ICIs demonstrated a significant correlation between LRGs with ICI markers including, PD-1, PD-L1, and CTLA-4, except for *ALB*. Moreover, Single-Sample Gene Set Enrichment Analysis (ssGSEA) confirmed that the *MKI67 and RACGAP1* are significantly associated with lactylation-related pathways, which are crucial in cancer development (71). Furthermore, the LASSO model identified key lactylation-related prognostic biomarkers for HCC, including *MKI67* and *RACGAP1* showed a significant predictor while ALB had the least impact. This analysis enhances our understanding of identified LRGs in HCC biology. Collectively, these findings suggest that the LRGs can play a role in immune response therapy strategies, but further studies and clinical trials are needed to assess their efficacy and safety.

Previous studies have reported that identifying biomarkers using genomic analysis significantly improves HCC treatment, yet need to identify more molecules for early diagnosis, and targeted therapies (48). Consistent with these studies, our study shows that LRGs significantly correlated with approved biomarkers like AFP, GPC3, and GP73. Thses results suggest their potential as early diagnostic targets. Further validatory studies are required to confirm these findings and explore the clinical applications.

In summary, we validate our bioinformatics findings by employing IHC analysis of HCC patients’ samples. The current study demonstrated that *MKI67* and *RACGAP1* are significantly overexpressed in HBV/HCV-related HCC, compared to HCC without viruses. This overexpression suggests a more significant role in the development of HCC induced by HBV/HCV. These results revealed that *MKI67* and *RACGAP1* can predict outcomes and serve as pivotal therapeutic targets, especially for HCC related to HBV/HCV. This research provides valuable insights into the distinctive features of HCC associated with HBV/HCV and paves the way for new diagnostic and therapeutic strategies.

While this study identified potential biomarkers and therapeutic targets by comprehensive bioinformatics analysis and validation with IHC. However, some limitations can be acknowledged. First, the data was taken from public databases, there may be variations in results due to differences in patient selection and data processing. Secondly, the sample size was limited, which may affect the generalizability of findings. Future prospective cohort studies with a significant number of patients should be conducted. Thirdly our study didn’t consider the several factors that can influence HCC progression including, smoking, alcohol drinking, diabetes, lifestyle, etc. These factors should be considered in future studies for better HCC outcomes. Further studies are needed to investigate the mechanism underlying these interactions and potential therapeutic applications.

## Conclusion

In conclusion, this study identified six lactylate-related genes (*ALB*, *G6PD*, *HMGA1*, *MKI67*, *RACGAP1*, and *RFC4*) as promising independent prognostic biomarkers for HCC. *MKI67* and *RACGAP1* were especially identified as a predictive signature with prognostic potential for HBV and HCV-related HCC. These genes provide insights into poor survival and immune cell infiltration in tumors. Validation using HCC patient samples highlighted that *MKI67* and *RACGAP1*are significantly overexpressed in HBV/HCV positive HCC compared to HCC without viruses. These findings indicate that these genes can work as a potential biomarker for early diagnosis, management, and treatment of HCC caused by HBV/HCV via lactylation.

## Data Availability

The original contributions presented in the study are included in the article/**Supplementary Material**. Further inquiries can be directed to the corresponding authors.
