# Supplementary material for: RACGAP1 and MKI67 are potential prognostic biomarker in hepatocellular carcinoma caused by HBV/HCV via lactylation
Source: Front Oncol. 2025 May 12;15:1537084. doi: 10.3389/fonc.2025.1537084 (PMC12104089; doi:10.3389/fonc.2025.1537084)
Supplement: Supplementary file 1 [file DataSheet1.pdf]

Supplementary File-S1

Supplementary File-S2

Both files are attached as an Excel file with separate sheet numbers “Supplementary File S1-S2”

Supplementary File-S3

Attached is an Excel file for GO and KEGG output, as “Supplementary File S3”

Supplementary-S8-I

Figure attached S8-I: attached below

Supplementary-S4-A, B

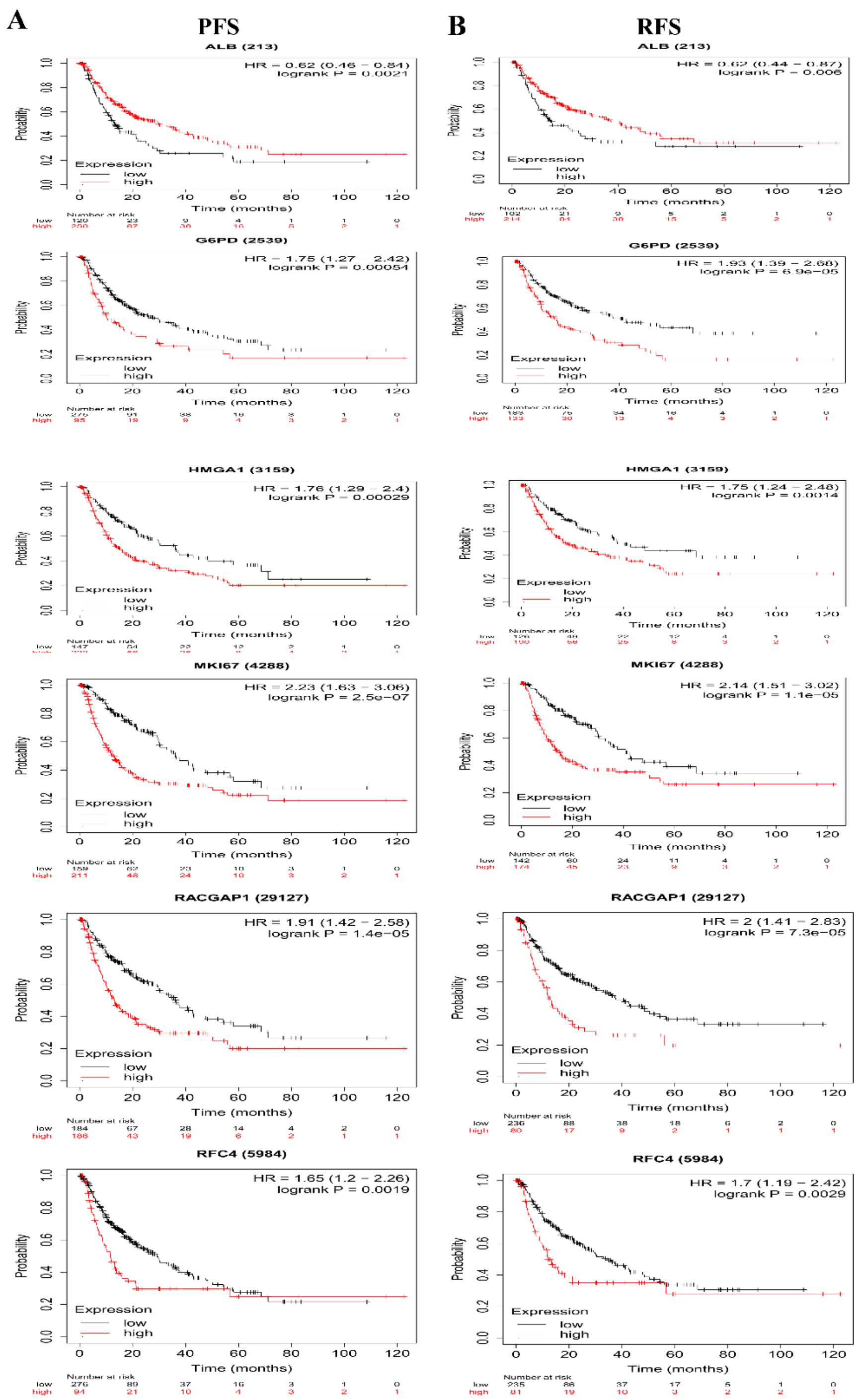

Supplementary-S4: Survival Analysis of six LRGs (A) Kaplan-Meier progression-free survival plots (B) Kaplan-Meier Recurrence-Free survival plots. Patients are categorized into high and low-expression groups based on the median gene expression levels.

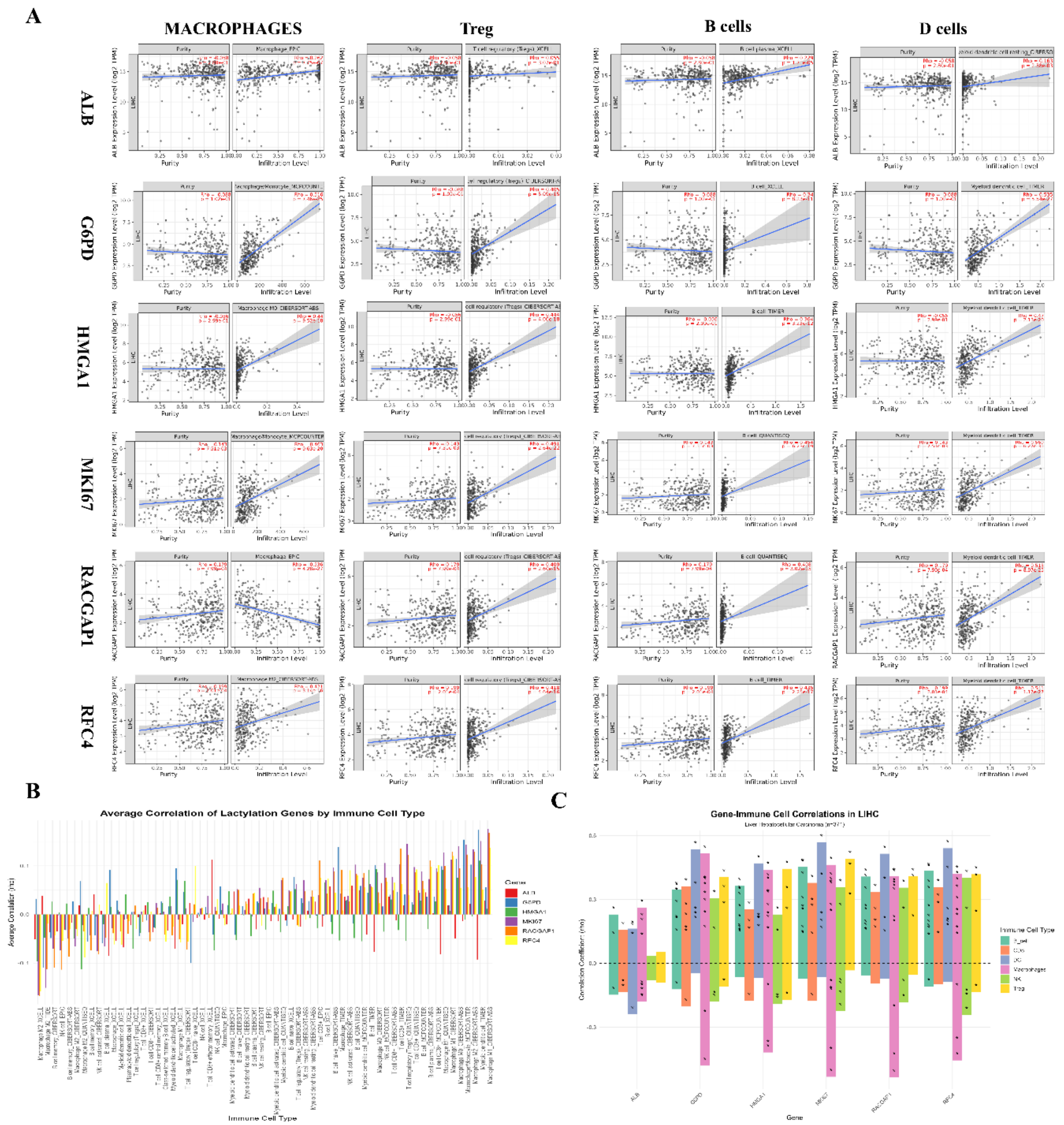

Supplementary-S5: (A) Correlation analysis of six lactylation-related gene expression and infiltration levels of Immune cells (Myeloid dendritic cells, Treg, B-cell, and Macrophages) (B) Bar plot illustrating Average correlations among lactylation-related genes across various immune cell infiltration levels. This plot summarizes the overall trends in gene-immune cell relationships across cancer types. (C) Bar plot illustrating mean correlations among lactylation-related genes across selected 06 immune cells. This plot summarizes the overall trends in gene-immune cell relationships with LIHC.

Supplementary-S6:

Additional files for Drug-interactions Attached is an Excel file "Supplementary File S6"

Also, add figure "**Supplementary-S6"**

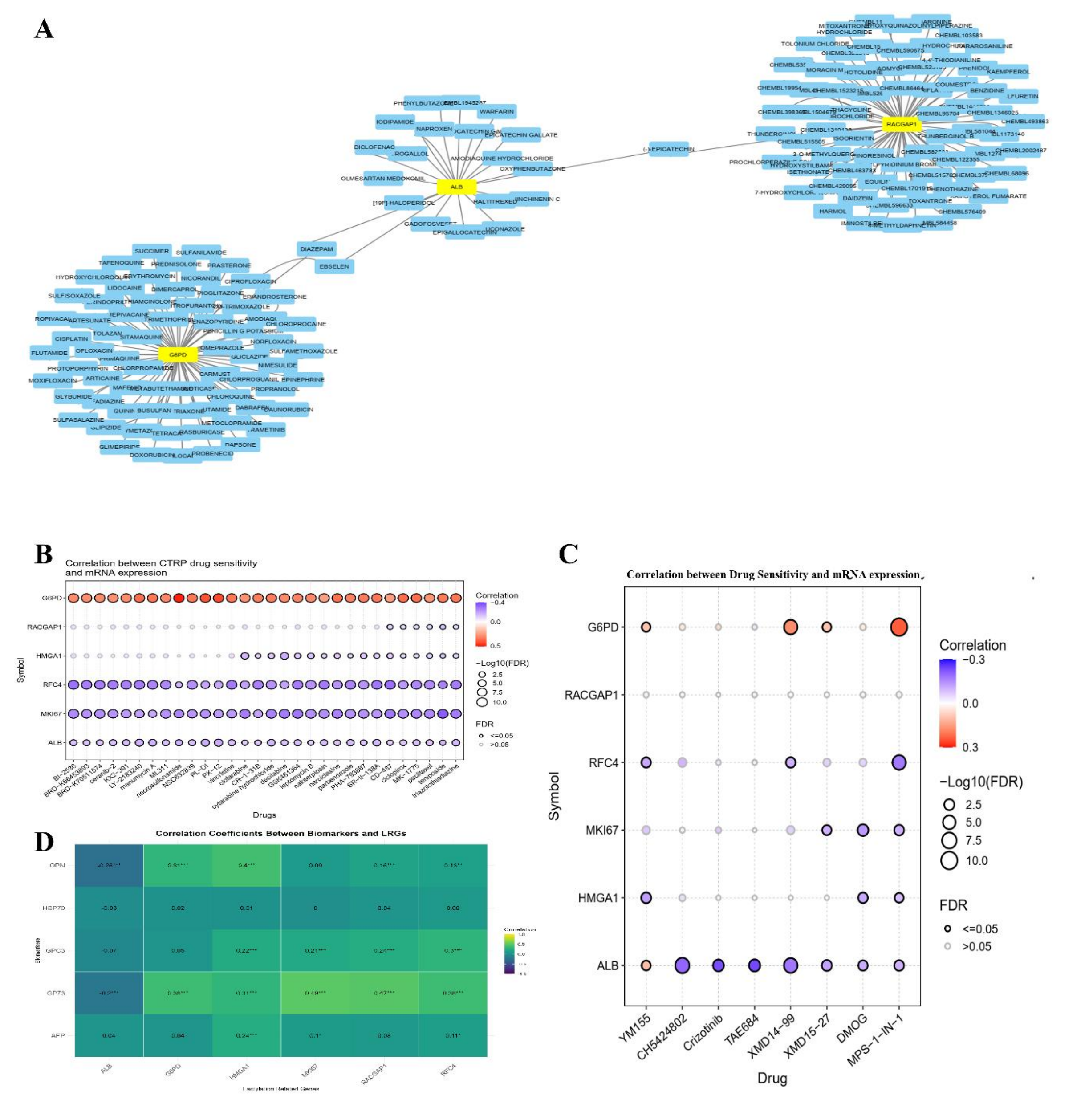

Supplementary-S6: (A) Network plot showing the interaction of drugs with identified LRGs (B) A correlation between CTRP drug and mRNA expression (C) Correlation of drug sensitivity and mRNA expression (D) A heatmap showing the correlation between well-known biomarkers and LRGs by using coefficient correlation methods.

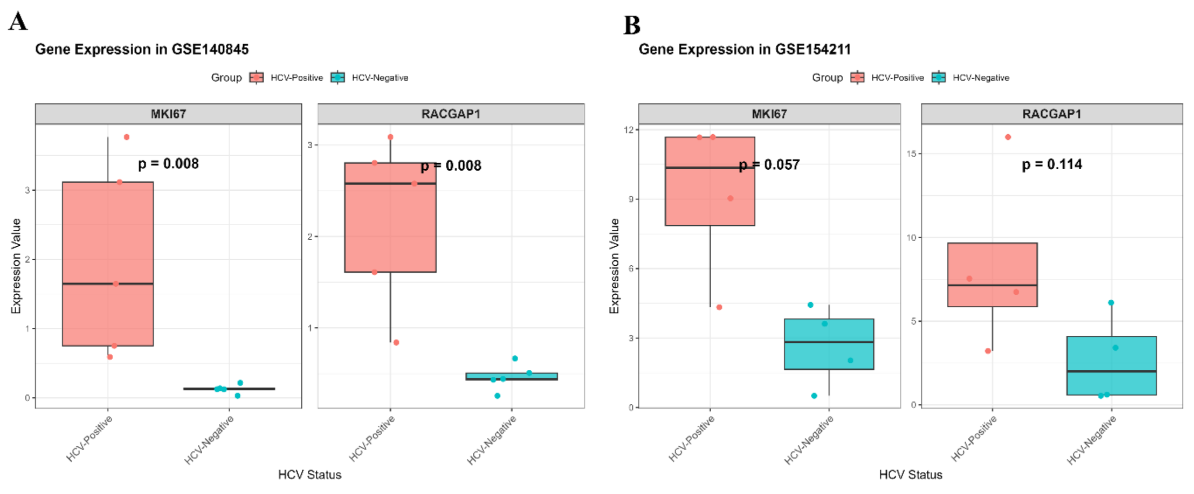

Supplementary-S7: (A) The box plots indicating the TPM expression value of *MKI67* and *RACGAP1* in GSE140845 with HCV-HCC vs HCC without HCV (B) Similarly, the box plots representing the TPM expression value of *MKI67* and *RACGAP1* in HCV-HCC vs HCC without HCV by using GSE154211 data. These findings confirmed the higher expression of *MKI67* and *RACGAP1* in HCC induced by HCV as compared to HCC without viruses.

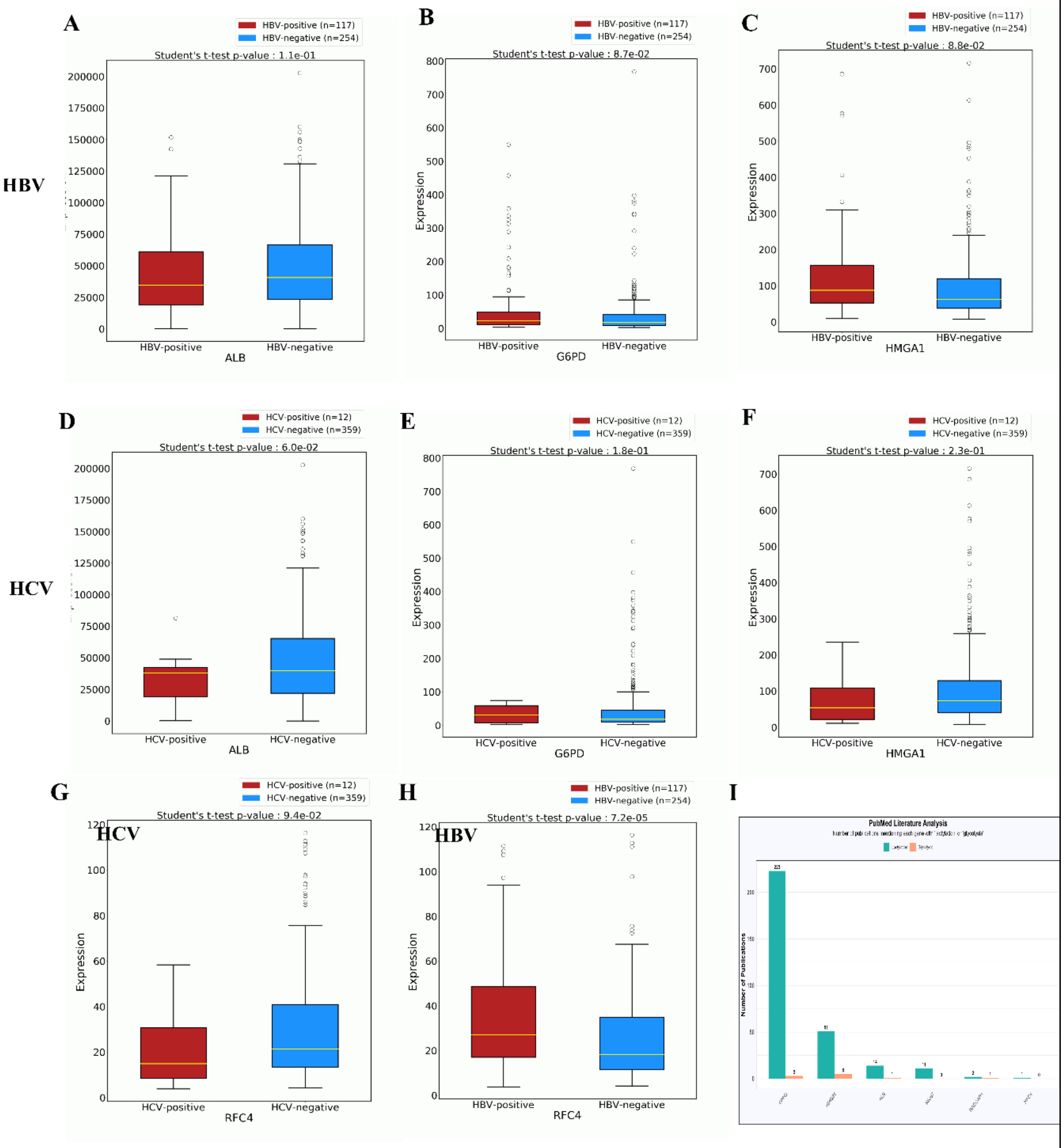

Supplementary-S8: (A-C) Box plot demonstrating the correlation of ***ALB, G6PD,*** and ***HMGA1*** expression with HBV-associated HCC patients. (D-F) Box plot illustrating the correlation of ***ALB, G6PD, HMGA1,*** and ***RFC4*** expression with HCV-associated HCC patients. (G-H) Box plots showing the correlation of *RFC4* with HCV, HBV with HCC (I) The bar plot indicating the available literature on PubMed
